# The relationship between subjective socioeconomic status and health in adults with and without intellectual disability

**DOI:** 10.1111/jar.13028

**Published:** 2022-08-24

**Authors:** Martin McMahon, Chris Hatton, Claire Hardy, Nancy J. Preston

**Affiliations:** ^1^ Division of Health Research Lancaster University Lancaster UK; ^2^ Health and Community Services Government of Jersey Jersey UK; ^3^ Faculty of Health, Psychology & Social Care Manchester Metropolitan University Manchester UK

**Keywords:** health, health inequalities, intellectual disability, MacArthur Scale of Subjective Social Status, socioeconomic status, subjective socioeconomic status

## Abstract

**Background:**

This study investigated if subjective socioeconomic status (SSS) is related to self‐rated health (SRH) and objective indicators of health in people with and without intellectual disability.

**Methods:**

Participants were 217 adults with, and 2350 adults without intellectual disability in Jersey. In the intellectual disability sample, 85 (39.2%) participants consented independently, while 132 (60.8%) participants consented through proxy procedures. The MacArthur Scale of Subjective Social Status was used to measure SSS. The Euro‐Qol EQ‐5D‐5L and a five‐point scale ranging from poor to excellent health were used to measure SRH.

**Results:**

Higher SSS and younger age were predictors of better SRH for the proxy‐report intellectual disability group. Being employed was associated with higher EQ‐5D‐5L index values for all intellectual disability groups.

**Conclusion:**

As SSS was only related to SRH in the proxy intellectual disability group, further research with a larger intellectual disability sample is needed to explore its utility further.

## 1 INTRODUCTION

People with intellectual disability have greater health needs (Hughes‐McCormack et al., 2018; McMahon & Hatton, 2021) and are more likely to die at a younger age than the general population (Glover et al., 2017; Landes et al., 2021; O'Leary et al., 2018). Such differences may be regarded as health inequalities (Emerson & Hatton, 2014). Health inequalities generally have strong associations with social and economic conditions (Marmot, 2005a, 2020; World Health Organisation [WHO], 2008) and a significant body of evidence has documented the association between these factors and health (Adler & Stewart, 2007; Dignan, 2001; Marmot et al., 1991; WHO, 2008). These factors known as social determinants of health are the non‐medical factors that influence health outcomes. For adults with an intellectual disability this is a complex area that is shaped by both internal and external conditions and the interplay between these (McMahon, 2022). For some people with an intellectual disability this is an important consideration as they are potentially more likely to be exposed to health inequalities from both a biological and non‐medical factor perspective. For example, regarding the concept of clinical phenotypes—which is the outward expression of genes—it is important to consider the manifestation of particular sets of physical problems commonly encountered with particular syndromes (for example Down syndrome and Alzheimer's type dementia) (Strydom et al., 2019). Additionally, people with intellectual disabilities are more likely to be disproportionally exposed to a cascade of disparities (Emerson & Hatton, 2014; Krahn & Fox, 2014; Marmot, 2005a) including unemployment (Hatton, 2018), poverty (Emerson, 2007; Emerson et al., 2006), exclusion (Merrells et al., 2018), low levels of education (McMahon et al., 2019), poorer access to healthcare (Krahn et al., 2015) and discrimination (Emerson, 2021).

Previous research on health inequalities has described societal gradients or social hierarchies existing within societies (Adler, 2009; Adler et al., 1994; Singh‐Manoux et al., 2003) suggesting a person's place on the gradient determines how long they will live and how healthy a life they will have (Marmot, 2020; Marmot et al., 2010). Traditionally, a person's place on this gradient has been determined by measuring their socioeconomic status. Conventional objective indicators of socioeconomic status include education, occupational status and income. The relationship between socioeconomic status and a person's health status is deeply patterned, with each affecting the other. The place a person is positioned on the gradient affects their health, and in turn, their health affects their capability to reach higher levels on this gradient. It is now accepted that socioeconomic status is the principal indicator of inequality where greater rates of morbidity and mortality are experienced amongst individuals who are at the lower end of this gradient (Adler, 2009; Cundiff & Matthews, 2017).

Although objective indicators of socioeconomic status are reliably associated with greater rates of mortality and morbidity (Donkin et al., 2018), evidence has suggested that subjective socioeconomic status (an individual's opinion of their rank within society, also referred to as subjective social status) is more strongly associated with a person's health than conventional objective socioeconomic status indicators (Euteneuer, 2014). Some researchers (Jackman, 1979; Singh‐Manoux et al., 2003) refer to a cognitive averaging process whereby subjective socioeconomic status is not only reflective of a person's socioeconomic position, but is a social phenomenon that captures a person's life chances, and other previous, current and future prospects that are independent of conventional objective measures of socioeconomic status. Substantial literature has considered the influence of subjective socioeconomic status on health, aligned to the notion that through individuals internalising their place within socioeconomic hierarchies, physiological stress‐related pathways are activated, negatively impacting a person's health (Marmot, 2005b; McEwen & Gianaros, 2010). Research has also found that material deprivation cannot alone account for all biological indicators of health status (Nobles et al., 2013) and low subjective socioeconomic status is associated with a higher prevalence of cardiovascular diseases (Allen et al., 2014; Marmot et al., 1991), respiratory diseases (Cohen et al., 2008), oral disease (Sanders et al., 2006), mental health problems (Demakakos et al., 2008) and obesity (Goodman et al., 2003).

Links between subjective socioeconomic status and health status have been reported in the UK (Singh‐Manoux et al., 2003; Singh‐Manoux et al., 2005), the USA (Franzini & Fernandez‐Esquer, 2006) and in ethnically diverse samples (Allen et al., 2014; Ostrove et al., 2000). Cundiff and Matthews (2017) identified that subjective socioeconomic status provides exclusive information for understanding health inequalities as it provides a unique cumulative association with physical health, particularly self‐rated health (SRH), exceeding conventional objective indicators of socioeconomic status. Theoretically, this has important implications for individuals with intellectual disabilities for two principal reasons. First, although SRH is under‐researched with people with intellectual disabilities (Emerson et al., 2014; Fujiura et al., 2012), it has notable predictive validity with respect to mortality in the general population (Schnittker & Bacak, 2014). Furthermore, the evidence that does exist suggests that poorer SRH may be the consequence of poorer living environments rather than a person's intellectual disability per se (Emerson et al., 2014). As far as we are aware there is no evidence to suggest that subjective socioeconomic status does not provide a unique cumulative association with physical health or SRH in the intellectual disability population similarly to the general population. Second, objective measures of socioeconomic status are potentially poor indicators in the intellectual disability population due to a lack of variation in these indicators; with uniformly low educational attainment, very low employment rates and low income in this group (Hatton, 2018). Similarly, indicators based on area deprivation around people's homes may be less relevant when people are living in residential care. Consequently, subjective socioeconomic status could be a more robust indicator for capturing the overall socioeconomic position of individuals with intellectual disabilities.

The literature on subjective socioeconomic status focuses on the MacArthur Scale of Subjective Status (Adler et al., 2000; Goodman et al., 2001; Ostrove et al., 2000; Singh‐Manoux et al., 2003). This is the principal measure used to capture an individual's perceived position within society. The MacArthur Scale of Subjective Social Status uses a ‘social ladder’ aligned to the social gradient within society and asks a respondent to rate the rung on which they feel they stand. The MacArthur Scale of Subjective Social Status was developed by Adler and Stewart (2007) and grounded in Cantril's (1965) earlier work investigating happiness using a similar self‐report ladder. Aligned to the societal hierarchy, the MacArthur Scale of Subjective Status summarise an individual's sense of their place on this ladder using a holistic self‐evaluation of socioeconomic status and social position. It appears to be a promising measure to determine the relationship between socioeconomic status and health status for people with intellectual disabilities, as it is potentially accessible and people with intellectual disabilities generally occupy atypical socioeconomic positions within society.

In a US based study, Queirós et al. (2015) used the MacArthur Scale of Subjective Status and identified that individuals with a cognitive disability did not rate their subjective social status as lower than their non‐disabled peers even though they had poorer educational attainment, occupational status and income. Whilst Queirós et al. (2015) do not explore this further, this theoretically reflects adaptation to the persistent deprivation that these individuals experience. This phenomenon is supported by quality‐of‐life research (Hensel et al., 2002) showing that individuals with intellectual disabilities may self‐report higher ratings on quality of life measures as they compare their own situation to other people with more severe intellectual disabilities (Simões et al., 2015; Stancliffe, 1999). Similarly, people with intellectual disabilities may have more of a positive outlook (Hartley & MacLean Jr, 2006) and may be less analytical of their environmental conditions (Perry & Felce, 2005). Considering this, the MacArthur measure for assessing subjective socioeconomic status may have applied benefits for research with people with intellectual disabilities for two primary reasons. First, the ladder is relatively cognitively unchallenging, and therefore inclusive for most individuals with intellectual disabilities. Second, it measures a complex phenomenon allowing for individuals to include subtle subjective indicators of health and wellbeing alongside self‐assessed objective indicators. This suggests that it is theoretically a robust measure to tease out where individuals position themselves on the socioeconomic hierarchy.

Given the substantial evidence for a positive association between subjective socioeconomic status and health in the general population, we are aware of no evidence that pertains to the intellectual disability population and its association with health. Understanding the interplay between this is an important consideration that needs prioritising given the atypical socioeconomic position that many people with intellectual disability occupy in society. Therefore, the aim of this study is to determine if subjective socioeconomic status is related to self‐rated and objective indicators of health in people with and without intellectual disability in Jersey.

## 2 METHODS

### 2.1 Context

This study was undertaken in Jersey, Channel Islands, a self‐governing British Crown dependency with a population of just over 105,000 (States of Jersey 2019). Jersey has a highly developed economy and a quality‐of‐life index of 163.35 (Europe range: Russia 101.67—Switzerland 190.82) (Numbeo, 2021). While employment has been impacted due to the COVID‐19 pandemic, from 2015 to 2020 the labour market has grown across most sectors and in 2019, 90% of working age adults were economically active. The cost of living in Jersey is high, driven in part by the sizeable finance industry that exists. For example, average earnings for full time workers range from £1080 per week in financial services to around £410 per week in hotels, restaurants and bars (Government of Jersey, 2020). This impacts consumer prices which are 31% (excluding rent) or 49% (including rent) higher than in the UK (Numbeo, 2021). The proportions of individuals living in ‘relative low income’ in Jersey, where they are living in households with an income below 60% of the median in that year has been stable over the last 10 years standing at approximately 22% (Government of Jersey, 2020). This is, however, greater than the UK where ‘relative low income’ stood at 16% in 2020/2021 (Francis‐Devine, 2022). No data exist regarding the proportion of people with an intellectual disability living in ‘relative low income’ in Jersey. However, a study by McMahon et al. (2019) describes a negative picture where they cite that the majority of people with an intellectual disability in Jersey have low levels of employment, poor income and rely on government benefits which are often aligned to physical and personal care needs. Homeownership is also low in Jersey with only 54% of people owning their own home in the last census (Government of Jersey, 2011); this compares to 63% of households in England owning their own homes in the 2 years from 2016 to 2018 [www.gov.uk, 2020]. The health of the Jersey population compares favourably to other developed countries and the leading causes of mortality (cancers and heart disease) are broadly similar to other developed countries (Government of Jersey, 2016). The health of people with intellectual disability in Jersey is poorer than the general population (McMahon & Hatton, 2021), similar to other developed countries (Emerson et al., 2014; Emerson & Hatton, 2014; Hughes‐McCormack et al., 2018; van Schrojenstein Lantman‐de Valk, 2005).

### 2.2 Ethics statement

Ethical approval was granted from the Faculty of Health and Medicine Research Ethics Committee at Lancaster University (reference FHMREC16083) and by the Government of Jersey, Health and Community Services Ethics Committee. The consent process and accompanying documentation was designed using guidance from the Mental Capacity Act (2005) and the Health Research Authority (https://www.hra.nhs.uk/). Further details of the consenting procedure for adults with an intellectual disability are outlined in Bowring (2017), McMahon et al. (2019), McMahon et al. (2020), Bowring et al. (2017a) and Bowring et al. (2017b).

### 2.3 Procedure

This was an original study and the structured survey instrument was specifically designed to collect data from people with and without intellectual disability in Jersey.

### 2.4 General population sample

After accounting for population density and excluding addresses that had previously been sent the 2015, 2016 or 2017 Annual Social Surveys, or the Disability Survey in 2015, 8000 surveys (weighted in terms of population density strata for each parish) were sent to households across the 12 parishes in Jersey. To account for the entire adult population at random, the household member who next celebrated their birthday, and who was aged 18 years or over, was asked to complete the survey. A total of 2415 surveys (30.2%) (age range 19–105, mean = 57.67, SD = 16.3) were returned with 65 of these being unusable. There was less than 2.5% missing data on any variable (range 0.8%–2.3%).

### 2.5 Intellectual disability sample

At the time of data collection, 285 adults were known to access intellectual disability services in Jersey. To access intellectual disability services in Jersey, individuals are assessed against three criteria by health and social care professionals. These criteria include significant limitations in intellectual functioning and adaptive behaviour with an onset before the age of 18. Individuals were asked to participate independently or where they lacked capacity they were consented through proxy procedures with the person and/or a personal or nominated consultee (Department of Health, 2008). The 217 adults with an intellectual disability who participated represented a 76% response rate. All information was collected by face‐to‐face interviews with participants or through proxy respondents. The proxy respondent was the person who knew the participant best and respondents included family members, key workers and friends. Eighty‐five (39.2%) participants consented independently, while 132 (60.8%) participants were consented through proxy procedures.

### 2.6 Subjective socioeconomic status

Subjective Socioeconomic Status was measured using the MacArthur Scale of Subjective Social Status (Adler & Stewart, 2007) (SSS ladder herein). Standard wording that accompanies the MacArthur Scale of Subjective Social Status was used to ask both populations of participants or proxies. For example: ‘Think of this ladder as showing where people stand in Jersey. At the top of the ladder are the people who are best off – those who have the most money, the best education, and the most respected jobs. At the bottom are the people who are worst off – those who have the least money, the least education, and the least respected job or no job. The higher up you are on this ladder, the closer you are to the people at the top; the lower you are, the closer you are to the people at the bottom’.

- Where would you place yourself (*or person you are answering on behalf of if proxy*) on this ladder?
- Place an ‘X’ on the rung where you think you (*or person you are answering on behalf of if proxy*) stand at this time of your life relative to other people in Jersey.

### 2.7 Objective socioeconomic status

Education, occupation and income were used as objective indicators of socioeconomic status. These variables along with other sociodemographic variables were collected to mirror the general population ‘Jersey Opinions and Lifestyle Survey’ (States of Jersey, 2017) and therefore were reflective of the educational and occupational landscape at the time of data collection. Education was categorised as; no formal education, GNVQ/BTEC Introductory Diploma (Foundation), ‘O' levels/CSE/GCSE/ BTEC First/ GNVQ (Intermediate), AS‐Level, /A2‐Level/BTEC National/GNVQ (Advanced), First Degree, Higher Degree (e.g., Masters/PhD) or other. Occupation was categorised as; working for an employer, self‐employed, not employing others, unable to work because of long‐term sickness or disability, unemployed, looking for work, unemployed, not looking for work, in full‐time education, a homemaker, retired or other. Individual income was categorised as income less than £15,000, increasing in £10,000 increments to income above £105,000.

### 2.8 Health

To measure SRH, participants or proxies were asked if their health was ‘excellent, very good, good, fair or poor’. The EQ‐5D‐5L EuroQol questionnaire was used to measure health‐related quality of life (HRQoL) across both populations (Devlin & Brooks, 2017). The EQ‐5D‐5L is a generic objective measure of health that comprises of a simple descriptive system and a visual analogue scale (VAS). The VAS is subjective in nature and comprises of a scale ranging from 0 to 100 asking respondents how they rate their health on the day of completing the questionnaire.

The descriptive element of this measure can be converted into a single summary index value from five dimensions of health: mobility, ability to self‐care, ability to undertake usual activities, pain/discomfort and anxiety/depression. These dimensions have five levels of severity for each dimension (no problems, slight problems, moderate problems, severe problems, and extreme problems). The present study used the corresponding English Crosswalk value set as advised by EuroQol for the EQ‐5D‐5L. This converts one of the different 3125 different health states into an index value ranging from −0.285 to 0.95, where −0.285 represents extreme problems on all dimensions and 0.95 represents full health (Devlin et al., 2018).

### 2.9 Sociodemographic variables

This study is part of a larger comparative study undertaken by the researchers and all demographic variables were collected to mirror the general population ‘Jersey Opinions and Lifestyle Survey’ (States of Jersey, 2017) that included variables such as gender, age and marital status.

### 2.10 Approach towards analysis

Data analysis was performed using the Statistical Package for the Social Sciences Version 25 (SPSS Inc., Chicago, IL, USA). Our approach to analysis was undertaken in six stages. First, due to the low variation and non‐normal distribution across populations, objective socioeconomic status indicators for adults with intellectual disabilities were recoded from ordinal and scale variables into binary variables. Education was recoded as ‘formal education vs no formal education’, income was recoded as ‘above or below £15,000 per annum’ and occupation was defined as ‘in employment vs unemployed’. Given the high number of retired respondents in the general population sample, we only analysed respondents in the occupation variable who identified as working for an employer, self‐employed, employing others, self‐employed, not employing others, unemployed, unable to work because of long‐term sickness/disability, unemployed, looking for work, or unemployed not looking for work. Self‐rated health was also recoded into a binary variable that represented ‘good to excellent’ health (excellent, very good and good) or poor health (fair or poor).

Second, we used descriptive statistics to describe the objective (education, occupation and income) and subjective socioeconomic status (SSS ladder) and health (EQ‐5D‐5L index values, visual analogue scale [0–100] and dichotomised self‐rated health [good to excellent vs poor to fair SRH]) of all three groups of respondents (general population, intellectual disability—self report and intellectual disability—proxy report). Third, error line graphs with 95% confidence intervals were used to graphically represent the variability of mean SSS ladder scores of all three groups stratified by age, SRH, employment, income and education. Fourth, inferential statistics aligned to the distribution of data (for example, chi‐square, Kruskal–Wallis *H* test, Mann Whitney *U* Tests, *t*‐tests and ANOVAS with Hochberg post hoc tests) to compare health by objective and subjective socioeconomic status.

Fifth, we used binary logistic regression to examine the association of subjective and objective socioeconomic status and demographic characteristics with SRH (good to excellent vs. poor to fair SRH) in people with and without intellectual disability. Finally, multiple regression using the stepwise procedure was used across stratified groups to determine the relationship between subjective and objective socioeconomic status and demographic characteristics with EQ‐5D‐5L index values. The stepwise procedure is an iterative construction of a regression model that involves the selection of independent variables to be used in a final model. Statistical significance was accepted at the ≤0.05 level of probability in all analysis.

## 3 RESULTS

Demographic and bivariate associations between personal characteristics, living circumstances, and indicators of socioeconomic status are presented in Table 1. Individuals with intellectual disability who self‐reported were older than people with proxy respondents but younger than the general population. All individuals with intellectual disability were more likely than the general population to have no formal education (*p* < .001), be unemployed (*p* < .001), and have an income of less than £15,000 (*p* < .001).

**TABLE 1:** Demographic, objective and subjective socioeconomic status characteristics of the general and intellectual disability populations

		General population *n*‐2350		Intellectual disability—Self report *n*‐85		Intellectual disability—Proxy report *n*‐132		*F*‐statistic	*p*
		57.65 (16.3)		39.2 (12.3)		47.9 (17.0)		72.38	<.001
Age (Mean, SD)		*n*	%	*n*	%	*n*	%	*χ* ^2^	*p*
Sex	Male	941	40.3	51	60.0	71	53.8	21.53	**<.001**
	Female	1394	59.7	34	40.0	61	46.2		
Degree of intellectual disability	Mild/moderate	–	–	84	98.8	80	60.6	3050.98	**<.001**
	Severe/profound	–	–	1	1.2	52	39.4		
Education	No formal education	498	21.5	67	78.8	127	96.2	468.29	**<.001**
	Formal education	1817	78.5	18	21.2	5	3.8		
Occupation	Employed	1371	94.4	31	43.7	12	10.8	732.16	**<.001**
	Unemployed	82	5.6	40	56.3	99	89.2		
Income	Under £15,000	476	22.0	65	82.3	122	94.6	438.46	**<.001**
	Above £15,000	1689	78.0	14	17.7	7	5.4		
		*n*	Median (IRQ)	*n*	Median (IRQ)	*n*	Median (IRQ)	*χ* ^2^	*p*
SSS Ladder Median (IRQ)		2350	6 (4,7)	82	4 (2,6)	131	3 (2,5)	110.51	**<.001**

*Note*: Bold value indicates statistical significance.

People with intellectual disability were more likely to self‐report ‘poor to fair’ SRH than the general population (general population ‘good to excellent’ 79.9% versus ‘poor to fair’ 20.1%; intellectual disability self‐report ‘good to excellent’ 72.9% versus ‘poor to fair’ 27.1%; intellectual disability proxy report ‘good to excellent’ 66.7% versus ‘poor to fair’ 33.3%) (*χ* ^(2)^ = 15.26, *p* < .001). No statistically significant difference was observed between the EQ‐5D‐5L index values for the general population and the intellectual disability self‐report group; however, the intellectual disability proxy‐report group had statistically significant lower index values than the self‐report group and general population (*p* < 0.001). In the VAS scores, while there were no differences between the intellectual disability groups, both the intellectual disability groups had significantly lower scores than the general population (*p* < .001) (Table 2).

**TABLE 2:** Self‐rated health, EQ‐5D‐5L index values and the distribution of EQ‐5D‐5L dimension responses for the general and intellectual disability populations

Self‐reported health	General population *N* (%)	Intellectual disability—Self report *N* (%)	Intellectual disability—Proxy report *N* (%)	Test statistic *χ* ^2^	*p*‐value
Good to Excellent SRH	1862 (79.2%)	62 (72.9%)	88 (66.7%)	15.26	**<0.001**
Poor to Fair SRH	467 (19.9%)	23 (27.1%)	44 (33.3%)		
**EQ‐5D‐5L index values with SPSS using the United Kingdom (UK) value set**	***N***	**Minimum/maximum**	**Mean (SD)**	**Test Statistic *F***	***p*‐value**
General Population	2316	−.43–1.0	0.80 (0.20)		
Intellectual Disability Self Report	85	0.02–1.0	0.80 (0.18)	72.121	**<0.001** ^**a**^
Intellectual Disability Proxy Report	129	−0.39–1.0	0.58 (0.35)		
**Visual Analogue Scale (0–100)**	**General population**	**Intellectual Disability**—**Self Report**	**Intellectual Disability**—**Proxy Report**	**Test Statistic *F***	***p*‐Value**
**Mean (Standard Deviation)**	77.14 (19.01)	70.74 (24.29)	70.27 (20.89)	11.92	**<0.001** ^**b**^
**Mobility**	**General Population *N* (%)**	**Intellectual Disability—Self Report *N* (%)**	**Intellectual Disability—Proxy Report** ***N* (%)**	**Test Statistic df (2)** *χ* ^2^	***p*‐value**
No problems	1694 (72.1%)	60 (70.6%)	68 (51.5%)		**<0.001**
Slight problems	331 (14.1%)	9 (10.6%)	16 (12.2%)		
Moderate problems	203 (8.6%)	11 (12.9%)	16 (12.1%)	39.696	
Severe problems	91 (3.9%)	4 (4.7%)	9 (6.8%)		
Unable to walk about	12 (0.5%)	1 (1.2%)	23 (17.4%)		
Self‐care					
No problems	2155 (91.7%)	72 (84.7%)	40 (30.3%)		**< 0.001**
Slight problems	105 (4.5%)	10 (11.8%)	30 (22.7%)		
Moderate problems	47 (2.9%)	2 (2.4%)	26 (19.7%)	476.421	
Severe problems	14 (0.6%)	0	9 (6.8%)		
Unable to wash or dress	13 (0.6%)	1 (1.2%)	27 (20.5%)		
Usual activities					
No problems	1672 (71.1%)	60 (70.6%)	77 (58.3%)		**0.001**
Slight problems	392 (16.7%)	19 (22.4%)	23 (17.4%)		
Moderate problems	196 (8.3%)	2 (2.4%)	19 (14.4%)	13.010	
Severe problems	44 (1.9%)	4 (4.7%)	10 (7.6%)		
Unable to do usual activities	30 (1.3%)	0	3 (2.3%)		
Pain/discomfort					
No pain/discomfort	907 (38.6%)	51 (60.0%)	76 (58.9%)		**< 0.001**
Slight pain/discomfort	928 (39.5%)	21 (24.7%)	29 (22.5%)		
Moderate pain/discomfort	397 (16.9%)	9 (10.6%)	17 (13.2%)	23.986	
Severe pain/discomfort	81 (3.4%)	4 (4.7%)	5 (3.9%)		
Extreme pain/discomfort	18 (0.8%)	0	2 (1.6%)		
Anxiety/depression					
Not anxious/depressed	1453 (61.8%)	43 (50.6%)	60 (45.5%)		**< 0.001**
Slightly anxious/depressed	590 (25.1%)	27 (31.8%)	35 (26.7%)		
Moderately anxious/depressed	230 (9.8%)	15 (17.6%)	27 (20.6%)	21.699	
Severely anxious/depressed	43 (1.8%)	0	5 (3.9%)		
Extremely anxious/depressed	15 (0.6%)	0	4 (3.1%)		

*Note*: Bold value indicates statistical significance.

^a^ There is no statistical difference between the general population and intellectual disability self‐report.

^b^ There is no statistical difference between the intellectual disability self‐report and intellectual disability proxy‐report.

**FIGURE 1:**
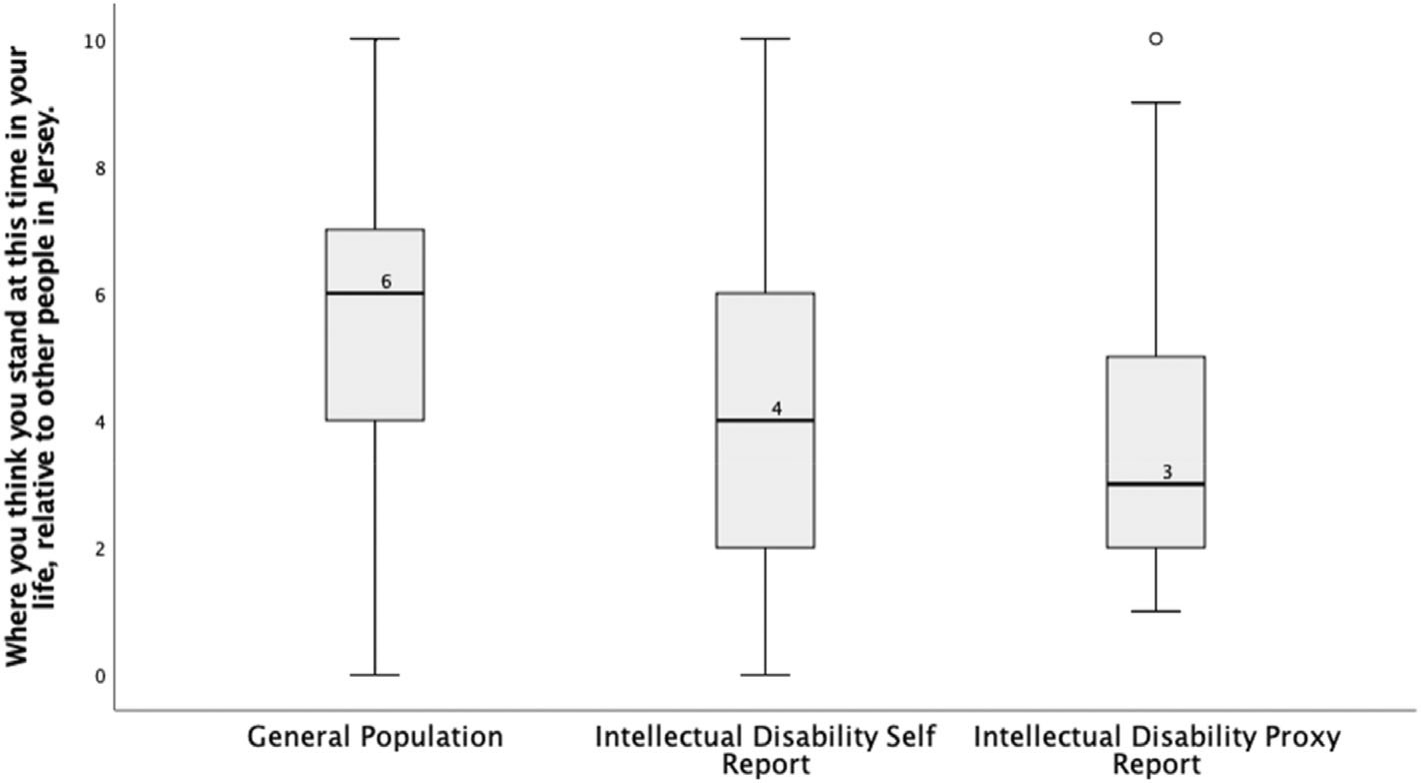
Boxplots presenting SSS ladder scores for the general and intellectual disability populations

Distributions of the SSS ladder scores for all groups are outlined in both Figure 1 and Table 1. There was a statistically significant difference in median between the different groups, *χ* ^2^(2) = 110.51, *p* < .001. This post‐hoc analysis revealed statistically significant differences in median scores between the general population (median (IQR) 6 (4,7)), the intellectual disability self‐report group (median (IQR) 4 (2,6), *p* = <.001) and the intellectual disability proxy‐report group (median (IQR) 3 (2,5), *p* < .001). No significant difference was observed between the two intellectual disability groups (*p* = .082).

**FIGURE 2:**
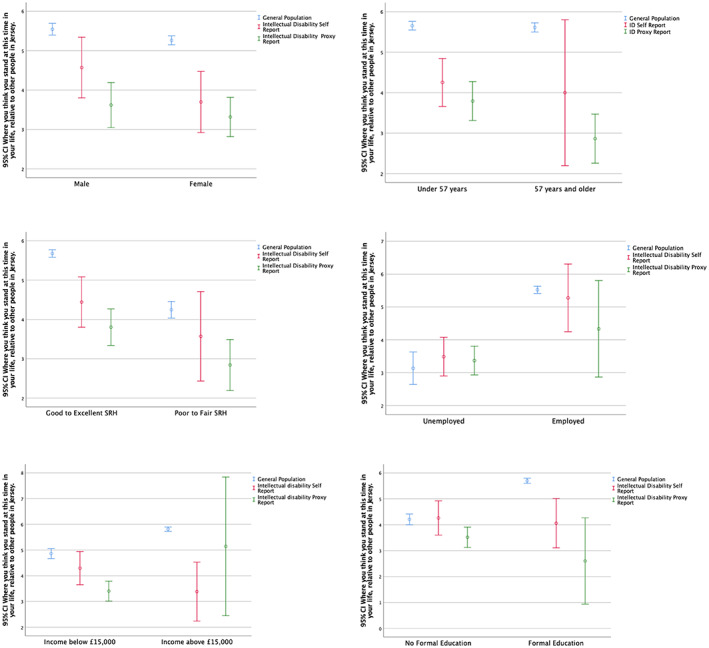
Stratified error line graph [95% confidence interval (CI)] representing the mean SSS ladder score by gender, age, self‐rated health, employment, income and education. *Note*: Error line graph is used to visualise the concentratedness of the SSS scores. People with an intellectual disability who self‐reported had higher mean SSS ladder scores for unemployment and no formal education than the general population. People with an intellectual disability who responded through proxy reporting had higher mean scores on the SSS ladder for unemployment and income below £15,000 than the general population.

The SSS ladder scores were stratified further to investigate the measure's relationship with, gender, age (split at median [less than or more than 57 years]) objective indicators of socioeconomic position (employment, education and income) and SRH (see Figure 2). Only older age (57 years or above) was associated with lower SSS ladder score in the intellectual disability self‐report population (*U* = 1420.500, *z* = −2.438, *p* = .015). Men had a higher SSS ladder score than women in the general population (*U* = 598,408, *z* = −3.612, *p* ≤ .001) but there were no statistically significant differences in SSS by gender in the intellectual disability populations *(p* ≥ .05). Being employed was associated with higher SSS ladder scores for both the general population (*U* = 24455.000, *z* = −8.704, *p* ≤ .001) and the self‐report intellectual disability population (*U* = 343.500, *z* = −2.778, *p* = .005) but not for the proxy report population (*p* = 0.133). Formal education (*U* = 275,672.500, *z* = −13.524, *p* ≤ .001) and income above £15,000 (*U* = 295,179.00, *z* = −8.961, *p* ≤ .001) were only associated with higher SSS scores in the general population. Good to excellent SRH was associated with higher SSS ladder scores in both the general population (*U* = 273,900, *z* = −12.520, *p* ≤ .001) and the proxy report intellectual disability population (*U* = 1339.00, *z* = −2.840, *p* = .005) but not in the self‐report intellectual disability population (*p* = .172). Additionally, there was a moderately positive significant correlation between SSS ladder scores and EQ‐5D index values in the general population, (*r* [2227] = .32, *p* < .0001) but not for any of the intellectual disability populations.

Binary regression analysis was conducted on the combined three groups. The model was statistically significant (*χ* ^2^(6) = 187.90, *p* < .0001) and indicated that higher SSS ladder scores, being employed and younger age were significantly associated with better SRH for the combined samples (data not shown). A second model was created that stratified the groups into ‘general population’ and ‘combined intellectual disability groups’. For the general population the effects of higher SSS ladder scores, being employed and younger age remained significant predictors of better SRH (see Table 3 Model 1) [*χ* ^2^(6) = 173.851, *p* < .0001]. However, for the combined intellectual disability group the effects of employment and SSS ladder scores attenuated, and younger age remained the only significant predictor of better SRH (Table 3 Model 2 = *χ* ^2^(6) = 16.203, *p* = .013). In the final model, the intellectual disability groups were further stratified into self‐report and proxy report groups. The self‐report group became non‐significant and all demographic, objective and subjective socioeconomic effects attenuated (data not shown as non‐significant). However, higher SSS ladder scores and younger age remained significant predictors of better SRH for the proxy‐report group (Table 3 Model 3) [*χ* ^2^(6) = 13.229, *p* = .040].

**TABLE 3:** Binary logistic regression analysis: associations between demographic, objective and subjective socioeconomic status and self‐rated health

	General population (Nagelkerke *R* ^*2*^ *.211)*					
Model 1	*β*	S.E.	Wald's *X* ^2^ (df 1)	Sig.	OR	95% CI for odds ratio
SSS Ladder	−.254	.039	43.453	**<.001**	.775	.719–.836
Income	.266	.246	1.172	.279	1.305	.806–2.113
Employment	−2.031	.309	43.100	**<.001**	.131	.072–.241
Education	−.219	.231	.901	.343	.803	.510–1.263
Age	.017	.008	5.032	**<.001**	1.017	1.002–1.033
Gender	.095	.174	.299	.584	1.100	.782–1.547
Constant	.496	.648	.587	.443	1.643	
Model 2	Combined Intellectual Disability Population (Nagelkerke *R* ^2^ *.127)*					
SSS Ladder	−.116	.084	1.909	.167	.890	.755–1.050
Income	−.327	.549	.355	.551	.721	.246–2.116
Employment	−.662	.506	1.709	.191	.516	.191–1.392
Education	.031	.708	.002	.965	1.032	.258–4.130
Age	.034	.012	7.753	**.005**	1.035	1.010–1.060
Gender	−.082	.361	.051	.821	.921	.454–1.869
Constant	−1.444	.918	2.475	.116	.236	
Model 3	Proxy‐report Intellectual Disability Population (Nagelkerke *R* ^2^ *.112*)					
SSS Ladder	−.223	.111	4.049	**.044**	.800	.644–.994
Income	−.768	.873	.774	.379	.464	.084–2.568
Employment	−.564	.856	.434	.510	.569	.106–3.048
Education	−20.13	28037.50	.000	.999	.000	.000‐.
Age	.031	.014	4.650	**.031**	1.031	1.003–1.061
Gender	−.151	.450	.112	.737	.860	.356–2.077
Constant	−.433	1.265	.117	.732	.649	

*Note*: Bold value indicates statistical significance.

Finally, multiple regression using the stepwise procedure using the EQ‐5D‐5L Crosswalk index value as the outcome variable was undertaken. Again, we stratified the groups into ‘general population’, ‘intellectual disability self‐report’ and ‘intellectual disability proxy report’. Results and test diagnostics considerations are outlined in Table 4. In summary, the final models predict that for the general population, people who are employed had higher EQ‐5D‐5L index values than those people who are unemployed, and an increase in one rung on the SSS ladder is associated with an increase in EQ‐5D‐5L index values. It also predicts that an increase in age by 1 year is predicted to decrease the EQ‐5D‐5L index values and earning less than £15,000 was associated with lower EQ‐5D‐5L index values. For the self‐report intellectual disability group, those who are employed have EQ‐5D‐5L index values that are higher than people who are unemployed and an increase in age of 1 year is also associated with lower EQ‐5D‐5L index values. For the proxy‐report intellectual disability population, that model predicted that people who are employed had EQ‐5D‐5L index values that are higher than people who are unemployed. No other significant associations were observed.

**TABLE 4:** Multiple regression using the stepwise procedure across the general and intellectual disability populations

EQ‐5D‐5L index value			Unstandardized coefficients		Standardised coefficients	95.0% confidence interval for B				
			B	Std. error	Beta	Lower bound	Upper bound	*R* ^2^	Δ *R* ^2^	Durbin‐Watson statistic
	Model							.237	.235	1.977
General Population	1	(Constant)	.498	.019		.461	.536			
		Employment	.346***	.020	.436	.308	.384			
	2	(Constant)	.446	.020		.407	.484			
		Employment	.304***	.020	.384	.266	.343			
		SSS Ladder	.017***	.002	.201	.013	.021			
	3	(Constant)	.515	.028		.461	.570			
		Employment	.297***	.020	.374	.258	.335			
		SSS Ladder	.017***	.002	.203	.013	.021			
		Age	−.001***	.000	−.085	−.002	−.001			
	4	(Constant)	.537	.030		.479	.595			
		Employment	.279***	.021	.352	.237	.321			
		SSS Ladder	.017***	.002	.196	.012	.021			
		Age	−.001***	.000	−.083	−.002	−.001			
		Income	−.031***	.014	−.057	−.060	−.003			
	Model							.149	.121	1.603
Intellectual Disability Self Report	1	(Constant)	.759	.030		.698	.819			
		Employment	.116*	.046	.304	.024	.209			
	2	(Constant)	.905	.079		.748	1.063			
		Employment	.120**	.045	.313	.029	.210			
		Age	−.004*	.002	−.237	−.008	.000			
	Model							0.094	0.085	1.428
Intellectual Disability Proxy Report	1	(Constant)	.519	.036		.448	.591			
		Employment	.351***	.107	.306	.138	.563			

*Note*: Model = ‘Stepwise’ method in SPSS; *R* 2 = coefficient of determination; Δ*R* ^*2*^ *= adjusted R*2.

* *p* < .05.

** *p* < .01.

*** *p* < .001.

## 4 DISCUSSION

In broad terms, our results indicate that adults with intellectual disability in Jersey are more likely to occupy lower socioeconomic positions than the general population with lower levels of education, employment and income. They are also more likely to report lower levels of SSS as measured on the MacArthur Scale of Subjective Social Status and lower SRH than the general population. For adults with intellectual disability who participated through proxy respondents, they were more likely to experience lower levels of health as measured by the EQ‐5D‐5L index value. For this group, employment was associated with better scores on the EQ‐5D‐5L index value. For self‐reporting adults with intellectual disabilities, employment and younger age were significant predictors of increased levels of health as measured on the EQ‐5D‐5L index value. Whereas for the general population, education, higher levels of SSS, younger age, and earning more than £15,000 were significant predictors of better health as measured on the EQ‐5D‐5L index value. Equally, for the general population, higher SSS, being employed and younger age were significant predictors of SRH. In contrast to these findings, higher SSS and younger age were only significant predictors of better SRH for the proxy‐report intellectual disability group.

These findings add to the existing evidence that individuals with intellectual disability have poorer SRH than the general population (Emerson et al., 2014) and are more likely to occupy low socioeconomic positions within society (Emerson & Hatton, 2014; Krahn & Fox, 2014). While the intellectual disability population had lower MacArthur SSS scores than the general population, this study found that SSS was associated with SRH in the proxy reported intellectual disability group, and likely to reflect people with greater intellectual disabilities. The relationship between SS and health held after accounting for demographic and objective socioeconomic status indicators in the general population; a finding consistent with international evidence (Präg et al., 2016). Notwithstanding this, it should be kept in mind that the self‐report intellectual disability sample was small in this study and the lower distribution of MacArthur scores would suggest that it would be sensible to undertake further research in larger intellectual disability samples. This is of particular importance as SSS offers the potential to reveal the effects of social hierarchy on health (Singh‐Manoux et al., 2005) given its association with a range of health markers and physical health, as well documented in the literature (Cundiff & Matthews, 2017; Singh‐Manoux et al., 2003; Singh‐Manoux et al., 2005).

Other considerations also need to be taken into account when determining the findings of this study, particularly when the relationship between SSS and SRH in the proxy report population is observed but not in the self‐report population. For example, the self‐reporting nature of what SSS means to people with an intellectual disability is an important deliberation. In the early examination of this area of research, Jackman and Jackman (1973) reported that SSS refers to the individual's perception of ‘his’ position in the social hierarchy. Therefore, it is theoretically plausible that due to social disconnectedness, isolation and other negative life events that this population often experiences (Amado et al., 2013; Emerson, 2021) many people with intellectual disability experience a social hierarchy that is shaped by limited and atypical life experiences and this may impact what SSS means for this population. This may be in direct contrast to the proxy respondents who may have an altogether different experience. This is worthy of further critique given that SSS largely represents the nuances of a person's social position (Adler et al., 2000; Adler & Stewart, 2007). Furthermore, as this is one of the first studies to use the MacArthur Scale of Subjective Social Status in a total population of adults with intellectual disability, the suitability of this measure needs further examination. While there is no question that people with an intellectual disability should be the primary source of comment on their perceived social status, opinions, feelings and thoughts (Kooijmans et al., 2022) and indeed this is well established as being the case (Emerson et al., 2013), in the general intellectual disability literature there remains a paucity of psychometrically sound self‐reporting measures (Vlissides et al., 2017) and this needs to be accounted for. It is therefore reasonable to conclude that further research is required to examine the psychometric properties of this measure to determine the reliability of the MacArthur Scale in this population.

Nevertheless, the results of the study also clearly highlight the importance of employment for all people. Being employed was a significant predictor of better health in this study over and above any other indicators for people with an intellectual disability. Although this supports the well‐established link between employment and health in the general population (Ross & Mirowsky, 1995) there is a very limited amount of research that has focused on health outcomes of employment for adults with intellectual disability (Dean et al., 2018). While both Robertson et al. (2019) and Emerson et al. (2018) have identified that the association between employment and better health is similar for adults with and without intellectual disabilities, the evidence is inconsistent. Conversely McGlinchey et al. (2013) identified that employment status was only significantly related with health status when no other variables were controlled for. When variables such as age, level of intellectual disability, gender and residence were considered, employment did not predict health status.

Additionally, while our results find a link between employment and better health, it is difficult to make inferences to determine if employment is a cause of better health, or a consequence of better health. That is to say, healthier people with intellectual disabilities are more likely to be in employment and employment also brings health benefits. Therefore, it is probably reasonable to conclude that remarkably little is known about this relationship in the intellectual disability population (Emerson, 2007) and therefore these results should be interpreted with caution.

Notwithstanding this, it is of particular interest that our study observed that of all of those unemployed, people with intellectual disability had higher mean scores on the SSS ladder scale than the general population. This may suggest that unemployment is a common socioeconomic disadvantage experienced by this population (McMahon et al., 2019) and consequently, it may not alter SSS ladder scores to the same as it did in the general population, thereby reinforcing the adaptation to persistent deprivation that these individuals may experience. Finally, for the intellectual disability self‐report group, younger age was associated with better health on the EQ‐5D‐5L. However, this needs to be considered from the perspective that people with intellectual disability are more likely than their peers to experience increased morbidities at a younger age (Heslop et al., 2014; McMahon & Hatton, 2021) and when considered through the lens that this sample was approximately 18 years younger than the general population, this may account for this difference.

## 5 LIMITATIONS

When considering these results the following six limitations need to be kept in mind; (1) these findings apply only to the administratively defined intellectual disability population in Jersey, while there may also be adults with intellectual disability not known to services who were not included; (2) the sample sizes are unequal and as can be observed from the results the magnitude of the differences between the medians across the intellectual disability populations for the SSS ladder is large. This is, in effect a result of the small sample size for the intellectual disability populations; (3) there was only a 30% response rate and there was a high number of respondents who were retired. However, it needs to be acknowledged that this is representative of the general population in Jersey; (4) as this study used two different methods to recruit participants, it is theoretically that people with an intellectual disability also completed the general population survey. To account for this, a variable was included in the survey to indicate if the returned survey was completed by someone with an intellectual disability. Nonetheless, given that general population cohort surveys are generally wholly exclusive for individuals with intellectual disabilities with greater needs, the methods used in this study were reasonable adjustments to include as many people as possible with intellectual disabilities; (5) the use of proxy subjective measure such as the SSS ladder is of questionable utility as a proxy measure and, (6) the psychometric properties of the SSS measure have not been examined in the intellectual disability population, and (6).

Notwithstanding these limitations, this is the first study that has considered the concept of subjective socioeconomic status in the intellectual disability population. Our results identify that while the SSS ladder shows promise, at this stage it is only related to SRH in the proxy intellectual disability group. Further research is needed to explore its utility further.

## FUNDING INFORMATION

The material in this study is based upon work supported by the Government of Jersey Health and Community Services and Les Amis Limited Registered Charity Jersey.

## CONFLICT OF INTEREST

The authors declare no conflict of interest.

## Data Availability

The data that support the findings of this study are available on reasonable request from the corresponding author. The data are not publicly available due to ethical restrictions.
